# First characterization of glucose flux through the hexosamine biosynthesis pathway (HBP) in *ex vivo* mouse heart

**DOI:** 10.1074/jbc.RA119.010565

**Published:** 2020-01-08

**Authors:** Aaron K. Olson, Bertrand Bouchard, Wei Zhong Zhu, John C. Chatham, Christine Des Rosiers

**Affiliations:** ‡ Division of Cardiology, Department of Pediatrics, University of Washington, Seattle, Washington 98105; § Seattle Children's Research Institute, Seattle, Washington 98101; ¶ Montreal Heart Institute Research Center and Department of Nutrition, Université de Montréal, Montreal, Québec H1T 1C8, Canada; ‖ Division of Molecular and Cellular Pathology, Department of Pathology, University of Alabama, Birmingham, Alabama 35294

**Keywords:** glucose, glucose metabolism, carbohydrate metabolism, cardiac metabolism, O-linked N-acetylglucosamine (O-GlcNAc), post-translational modification (PTM), glucosamine, hexosamine biosynthesis pathway, metabolic flux, protein glycosylation, UDP-GlcNAc

## Abstract

The hexosamine biosynthesis pathway (HBP) branches from glycolysis and forms UDP-GlcNAc, the moiety for *O*-linked β-GlcNAc (*O*-GlcNAc) post-translational modifications. An inability to directly measure HBP flux has hindered our understanding of the factors regulating protein *O*-GlcNAcylation. Our goals in this study were to (i) validate a LC-MS method that assesses HBP flux as UDP-GlcNAc (^13^C)-molar percent enrichment (MPE) and concentration and (ii) determine whether glucose availability or workload regulate cardiac HBP flux. For (i), we perfused isolated murine working hearts with [U-^13^C_6_]glucosamine (1, 10, 50, or 100 μm), which bypasses the rate-limiting HBP enzyme. We observed a concentration-dependent increase in UDP-GlcNAc levels and MPE, with the latter reaching a plateau of 56.3 ± 2.9%. For (ii), we perfused isolated working hearts with [U-^13^C_6_]glucose (5.5 or 25 mm). Glycolytic efflux doubled with 25 mm [U-^13^C_6_]glucose; however, the calculated HBP flux was similar among the glucose concentrations at ∼2.5 nmol/g of heart protein/min, representing ∼0.003–0.006% of glycolysis. Reducing cardiac workload in beating and nonbeating Langendorff perfusions had no effect on the calculated HBP flux at ∼2.3 and 2.5 nmol/g of heart protein/min, respectively. To the best of our knowledge, this is the first direct measurement of glucose flux through the HBP in any organ. We anticipate that these methods will enable foundational analyses of the regulation of HBP flux and protein *O*-GlcNAcylation. Our results suggest that in the healthy *ex vivo* perfused heart, HBP flux does not respond to acute changes in glucose availability or cardiac workload.

## Introduction

Glucose is primarily metabolized for energy production via glycolysis and glucose oxidation. Glucose is also converted to glycogen or can be utilized in non-ATP-generating pathways, such as the pentose phosphate pathway and the hexosamine biosynthesis pathway (HBP).^2^ The HBP leads to the synthesis of UDP-GlcNAc, which is the major substrate for protein glycosylation that occurs in the endoplasmic reticulum and Golgi. UDP-GlcNAc is also the substrate for *O*-GlcNAc transferase (OGT), which catalyzes the post-translational modification of serine/threonine protein residues by *O*-linked β-N-acetylglucosamine (*O*-GlcNAc). Differing from most protein post-translational modifications, *O*-GlcNAcylation is regulated by a single enzyme for attachment (OGT) and a single enzyme for removal (*O*-GlcNAcase). Protein *O*-GlcNAcylation is a dynamic and ubiquitous process that occurs predominantly in the cytosol and nucleus, affecting a diverse array of protein functions and transcriptional events (1–3). In the heart, protein *O*-GlcNAc levels rise in association with various pathologies, including hypertrophy, heart failure, and diabetic cardiomyopathy (4–9). Acute increases in protein *O*-GlcNAcylation are reported to be cardioprotective as they promote myocardial recovery during ischemia/reperfusion or trauma-hemorrhage (10–17). The impact of prolonged changes in protein *O*-GlcNAc levels on the myocardium are variable and may depend on the pathological process (18). Reducing OGT and protein *O*-GlcNAc levels during pressure overload hypertrophy causes functional decompensation, whereas elevated protein *O*-GlcNAc levels appears to cause cardiomyopathy during diabetes (4–9). Although protein *O*-GlcNAcylation has important effects in the myocardium and other tissues, our understanding of the factors regulating protein *O*-GlcNAc levels is limited.

It is commonly stated that 2–3% of glucose is metabolized via the HBP; however, this percentage was estimated from a single study in rat adipocyte cell cultures (19), and its wider relevance is questionable. Moreover, because this estimate is represented as a percentage of total glucose uptake, it is unclear whether this value would be similar between quiescent cells in culture or in metabolically active organs with a greater energy requirement, such as the heart. Despite the importance of the HBP, there have been no reports on the flux of glucose through the HBP in any biological system. Nevertheless, the link between glucose and protein *O*-GlcNAcylation represents a mechanism by which changes in metabolism could directly regulate protein and cellular function.

**Figure 1.**
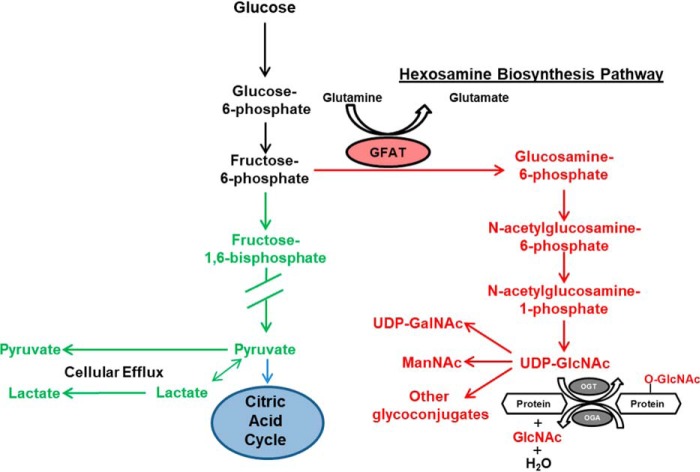
**Glycolysis and the HBP.** This scheme shows important metabolic intermediates for these pathways. Intermediates shared between glycolysis and HBP are shown in *black*, HBP-only intermediates are shown in *red*, and glycolysis-only intermediates are shown in *green*. Glucose is initially metabolized to G6P in the first glycolytic step. F6P, the next glycolytic intermediate, can either enter the HBP or undergo further glycolysis to generate pyruvate. GFAT (shown in the *red oval*) is the rate-limiting enzyme in the HBP and uses glutamine to catalyze the conversion of F6P to glucosamine 6-phosphate. After a series of reactions, uridine diphosphate-β-GlcNAc (UDP-GlcNAc) is the end product of the HBP and serves as the donor for the enzyme OGT to perform *O*-GlcNAc protein post-translational modifications. The enzyme *O*-GlcNAcase (*OGA*) removes the GlcNAc moiety from proteins. UDP-GlcNAc can also be epimerized to UDP-GalNAc, metabolized to *n*-acetylmannosamine (*ManNAc*), or utilized in other glycoconjugation reactions. Pyruvate is the end product of glycolysis and can enter the citric acid cycle, be metabolized to lactate, or be excreted from the cell (cellular efflux) as pyruvate or lactate.

At the level of fructose 6-phosphate (F6P), metabolized glucose can enter the HBP or continue through glycolysis to generate pyruvate (Fig. 1). l-Glutamine:fructose-6-phosphate amidotransferase (GFAT) is the rate-limiting enzyme in the HBP and uses glutamine to catalyze the conversion of F6P to glucosamine 6-phosphate (20), which is subsequently metabolized to UDP-GlcNAc, the end product of the HBP. OGT is sensitive to UDP-GlcNAc concentration, making overall protein *O*-GlcNAc levels responsive to changes in HBP flux (21). Consequently, it is widely accepted that changes in nutrient availability in general and more specifically glucose regulate HBP flux and protein *O*-GlcNAc levels; however, the data to directly support this remain surprisingly limited (16, 22–24). An inability to assess HBP flux has markedly hindered our understanding of the factors regulating protein *O*-GlcNAc levels in the heart and other tissues.

Accordingly, the goal of this study was to develop a method for quantifying glucose flux through the HBP. We used the *ex vivo* perfused working heart model, which is a commonly utilized experimental technique to quantify metabolic fluxes for energy production using ^13^C-labeled substrates and subsequent ^13^C-isotopomer analysis of the cardiac tissue (25). We first developed and validated a method for measuring UDP-GlcNAc ^13^C-enrichment and concentration using LC-MS. We chose LC-MS because of the high sensitivity and precision to assess tissue levels of unlabeled and ^13^C-labeled intermediates from both glycolysis and HBP without additional derivatization. Subsequently, we measured glucose flux through the HBP and determined whether altering glucose availability and/or workload affects HBP flux in the isolated perfused heart.

## Results

### LC-MS method development and characteristics

**Figure 2.**
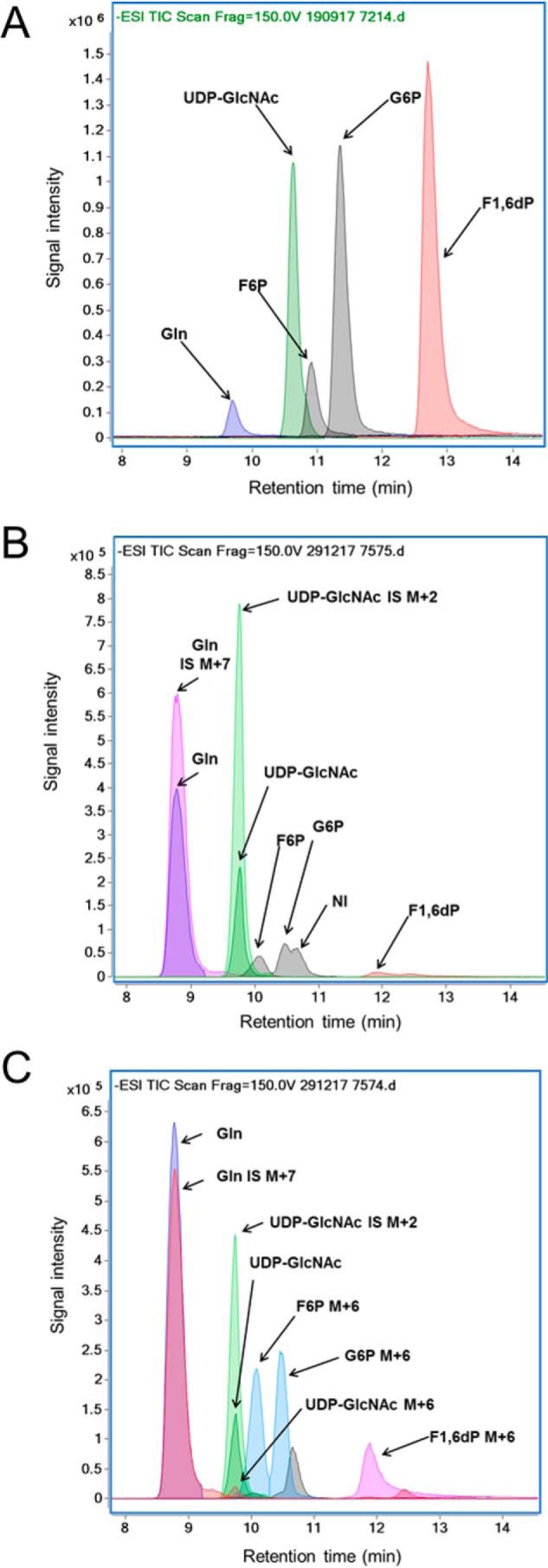
**Chromatographic separation using LC-QToF of UDP-GlcNAc, Gln, G6P, F6P, and F1,6dP in a representative sample of standard solution (*A*) and 50 mg of tissue extract from a heart without (*B*) or with (*C*) perfusion with 10 mm [U-^13^C_6_]glucose (MPE = 99%) added to 4 nm of [^13^C_2_]UDP-GlcNAc and 150 nm of [^13^C_5_,^15^N_2_]glutamine.** Signal intensity is shown for the extracted ion chromatograms at the following *m*/*z*: UDP-GlcNAc, 606.0743 (unlabeled), 608.0815 (M+2), and 612.0944 (M+6); Gln, 145.06119 (unlabeled) and 152.0727 (M+7); G6P, 259.0224 (unlabeled) and 259.0224 (M+6); F6P, 259.0224 (unlabeled) and 259.0224 (M+6); and F1,6dP, 338.9888 (unlabeled) and 345.0089 (M+6).

Our overall goal was to assess glucose partitioning between the HBP and glycolysis. We aimed to develop a LC-MS method that enables reproducible and quantitative measurements in heart tissue of unlabeled and ^13^C-labeled UDP-GlcNAc, which is the final HBP intermediate. In addition, we aimed to assess simultaneously using the same method (i) the tissue level of glutamine as well as (ii) unlabeled and ^13^C-labeling of intracellular metabolites shared or closely related to the HBP, namely the glycolytic intermediates glucose 6-phosphate (G6P), F6P, and fructose 1,6-diphosphate (F1,6dP; the metabolite immediately downstream of the regulator phosphofructokinase step of glycolysis) (see Fig. 1). Fig. 2 (*A* and *B*) shows representative chromatograms for all of these unlabeled metabolites in a standard solution (Fig. 2*A*) and plus the internal standards of [^13^C_2_]UDP-GlcNAc and [^13^C_5_-N_2_]glutamine in a nonperfused heart sample (Fig. 2*B*). Table 1 reports LC-quadrupole time-of-flight (LC-QToF) method characteristics, namely *m*/*z* and retention times, as well as intra- and interday coefficients of variation (%CVs) for the quantitative assessment of unlabeled UDP-GlcNAc and glutamine using their respective labeled internal standard, as well as semiquantitative assessment of G6P, F6P, and F1,6dP, using the external standard of [^13^C_2_]UDP-GlcNAc. Reported retention times are for heart tissue extracts, but note that they do slightly vary between experiments. There was very good reproducibility for all metabolites, as evidenced from the intra- and interday %CV, which were all below 15%, except for F1,6dP for which there was more variability (%CV = 27–30%) due to peak tailing. In 50 mg of heart tissues, the range of endogenous UDP-GlcNAc concentrations (0.7–3.4 nmol) is well above the limit of quantification (<0.01 nmol). Excellent linearity was observed for the measured level of all metabolites in the range of tested mg of heart tissues (20–70 mg; *R*^2^ > 0.94), although %CV values were better at 35 mg of heart tissue and above (data not shown). Of note, using this method, the heart tissue level of other HBP intermediates (namely glucosamine 6-phosphate, GlcNAc-6-phosphate, and GlcNAc-1-phosphate) were below our detection levels. Finally, short-term storage at room temperature of extracted samples resulted in no significant variation changes after 7 days upon vortex and sonication.

**Table 1:** **Characteristics of LC-QToF method for (semi)quantitative analysis of metabolites related to the HPB and glycolysis in heart tissues** Intraday and interday reproducibility for the (semi)quantification of metabolites were determined by analyzing eight homogenates of 50 mg of pulverized heart tissues added to 4 nmol of [^13^C_2_]UDP-GlcNAc and 150 nmol of [^13^C_5_-^15^N_2_]glutamine for 3 consecutive days. RT, retention time.

Analyte	*m*/*z*	Internal/external standard	*m*/*z*	RT	Intraday %CV	Interday %CV
				*min*		
UDP-GlcNAc	606.0743	[1,2-^13^C_2_]UDP-GlcNAc	608.0815	9.8	4.0	6.3
Glutamine	145.0619	[^13^C_7_]glutamine	152.0727	8.8	2.7	3.7
G6P	259.0224	[1,2-^13^C_2_]UDP-GlcNAc	608.0815	10.5	12.9	12.3
F6P	259.0224	[1,2-^13^C_2_]UDP-GlcNAc	608.0815	10.1	8.2	9.6
F1,6dP	338.9888	[1,2-^13^C_2_]UDP-GlcNAc	608.0815	11.9	27.3	29.9

### Pilot studies identifying heart perfusion conditions for ^13^C labeling of the final HBP intermediate UDP-GlcNAc

**Figure 3.**
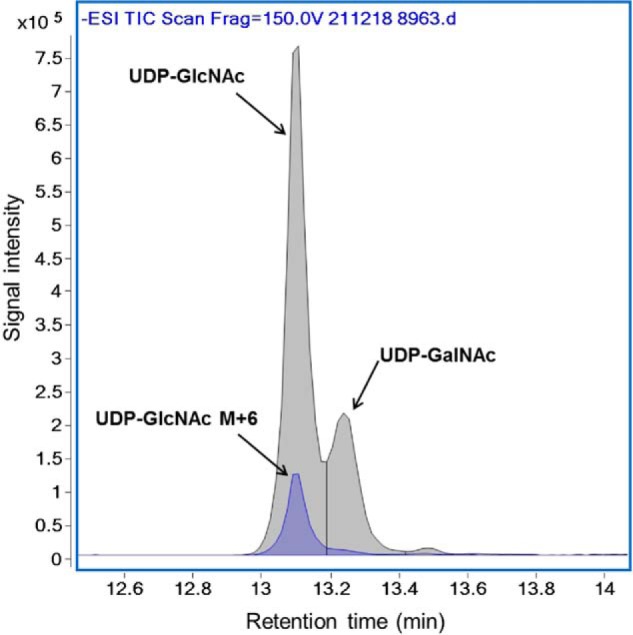
**Chromatographic separation using LC-QToF of UDP-GlcNAc *versus* UDP-GalNAc in a representative sample of 50 mg of tissue extract from a heart perfused with 5.5 mm [U-^13^C_6_]glucose (MPE = 99%).**

We first tested our LC-MS method for its capacity to accurately assess ^13^C-enrichment of UDP-GcNAc as well as G6P, F6P, and F1,6dP in tissues from rat heart that had been perfused with 5.5 mm [U-^13^C_6_]glucose (molar percentage enrichment (MPE) between 25 and 35%) that were part of a previous study (26). The M+6 MPEs, indicating the percentage of a metabolite generated from the exogenous [U-^13^C_6_]glucose, for G6P, F6P, and F1,6dP were at the expected values of around 25–35% (data not shown). Although there was a robust signal for unlabeled UDP-GlcNAc, the M+6 MPE was <2%, which is near lower detection limits. These results led us to perform additional experiments to validate the LC-MS measurement of ^13^C-labeled metabolites, particularly UDP-GlcNAc M+6 MPE, described in detail below, in which we use U-^13^C–labeled glucosamine or glucose (MPE = 99%). Fig. 2*C* shows a LC-QToF chromatograms for a representative analysis of a sample from hearts perfused with 10 mm [U-^13^C_6_]glucose (MPE = 99%). In this case, the signal intensity for G6P, F6P, and F1,6dP is almost exclusively due to M+6 (MPE = 96–97%); the level of unlabeled G6P, F6P, and F16dP is very low. For UDP-GlcNAc, the signal due to M+6 UDP-GlcNAc is still small compared with that of the unlabeled UDP-GlcNAc, but the M+6 MPE (12.6%) can now be assessed with precision. Of note, UDP-GlcNAc and UDP-GalNAc have similar chemical properties and could not be separated with the chromatographic conditions (described under “Experimental procedures”) utilized for the above results. Therefore, as part of our method development, we also assessed the percentage of UDP-GalNAc contributing to the UDP-GlcNAc peak using different chromatographic conditions (described under “Experimental procedures”) in nonperfused mouse hearts as well as in hearts perfused with [U-^13^C_6_]glucose (99%). The percentage of UDP-GalNAc to UDP-GlcNAc was similar between perfused and nonperfused hearts (*n* = 4 for each group) on average (11.1 ± 0.8% and 10.3 ± 1.1%, respectively). Fig. 3 shows a representative LC-QToF chromatogram for a sample of a heart perfused with 5.5 mm [U-^13^C_6_]glucose (MPE = 99%) that shows that the peak of UDP-GlcNAc, which is most abundant and is also predominantly ^13^C-labeled (M+6) (from experimental group “Beating”; see below for details). Given the relatively low level of UDP-GalNAc, we did not separate UDP-GlcNAc and UDP-GalNAc in our experiments, and values are reported as UDP-GlcNAc rather than UDP-HexNAc (made up of both UDP-GlcNAc and UDP-GalNAc).

### Evaluating the effect of working heart perfusions with [U-^13^C_6_]glucosamine on UDP-GlcNAC M+6 MPE and tissue concentration

Glucosamine is phosphorylated by hexokinase to form glucosamine 6-phosphate and enters the HBP after the rate-limiting enzyme GFAT. Supraphysiologic provision of glucosamine during working rat heart perfusions acutely increased total protein *O*-GlcNAcylation, presumably by increasing UDP-GlcNAc synthesis (27). To test our methods for measuring UDP-GlcNAc M+6 enrichment, we perfused mouse hearts in an *ex vivo* working mode with unlabeled physiological substrates and varying concentrations (0.001, 0.01, 0.05, or 0.1 mm) of [U-^13^C_6_]glucosamine (MPE = 99%) for 30 min (see “Experimental procedures” for details). The 0.001 and 0.01 mm [U-^13^C_6_]glucosamine were also perfused for 60 min.

**Figure 4.**
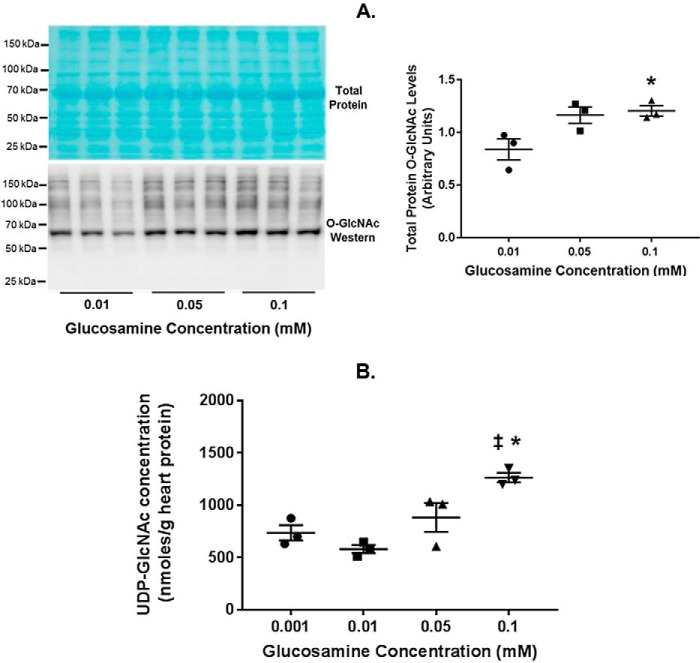
**Total tissue protein *O*-GlcNAc levels and UDP-GlcNAc concentrations in glucosamine working heart perfusions.** *A*, immunoblot for total protein *O*-GlcNAc levels in the indicated glucosamine concentrations. Total protein *O*-GlcNAc levels were normalized to the total protein stain (shown). *B*, UDP-GlcNAc concentrations normalized to heart protein in the indicated glucosamine concentrations. *Bars*, means ± S.E. (*error bars*); *n* = 3 for all groups. *, *p* < 0.05 between the indicated group and the 0.01 mm glucosamine concentration; ‡, *p* < 0.05 between glucosamine 0.1 and 0.001 mm. In *A*, *p* = 0.058 between 0.01 and 0.05 mm glucosamine.

Cardiac functional parameters (Table 2) during these perfusions are consistent with previous studies from our laboratory (28–31). The +d*P*/d*T*_max_, a measure of cardiac contractility, was slightly lower with glucosamine concentrations of 0.05 and 0.1 mm *versus* the other concentrations; however, none of the other functional parameters differed among the groups. We also evaluated the effect of glucosamine concentrations on myocardial total protein *O*-GlcNAc levels and UDP-GlcNAc concentration. Compared with 0.001 mm glucosamine, 0.05 or 0.1 mm glucosamine increased total protein *O*-GlcNAc levels, although this difference only reached significance (*p* value < 0.05) with 0.1 mm (*p* value was 0.058 with 0.05 mm) (Fig. 4*A*). Glucosamine (0.1 mm) also augmented UDP-GlcNAc concentration compared with the lower glucosamine concentrations (Fig. 4*B*), consistent with our prior study (27).

**Table 2:** **Working heart function during glucosamine perfusions** Values include both unlabeled and ^13^C-labeled glucosamine perfusions. Cardiac function is reported at 20-min perfusion time (both 30- and 60-min perfusion duration groups) and at 50-min perfusion time (60-min perfusions only). Values are mean ± S.E. *, *p* < 0.05 *versus* 20-min glucosamine (0.001 mm). #, *p* < 0.05 *versus* 20-min glucosamine (0.01 mm).

Glucosamine (mm)	20 min				45 min	
	0.001 (*n* = 12)	0.01 (*n* = 12)	0.05 (*n* = 6)	0.1 (*n* = 10)	0.001 (*n* = 5)	0.01 (*n* = 6)
Heart rate (beats/min)	372 ± 8	383 ± 10	406 ± 15	400 ± 17	392 ± 20	381 ± 16
Developed pressure (mm Hg)	71.5 ± 1.9	70.3 ± 2.4	67.7 ± 2.4	67.6 ± 2.1	72.4 ± 2.8	69.0 ± 1.4
+d*P*/d*T*_max_ (mm Hg/s)	4346 ± 147	4170 ± 180	3744 ± 184*	3650 ± 150*^#^	4435 ± 185	4092 ± 125
−d*P*/d*T*_min_ (mm Hg/s)	−3269 ± 112	−3183 ± 117	−3269 ± 227	−3144 ± 123	−3129 ± 77	−3053 ± 166
Aortic flow (ml/min)	11.2 ± 0.4	10.7 ± 0.5	9.9 ± 0.6	11.0 ± 0.3	10.6 ± 0.3	8.5 ± 1.1
Coronary flow (ml/min)	3.1 ± 0.2	3.3 ± 0.3	3.4 ± 0.2	3.1 ± 0.2	3.0 ± 0.2	4.1 ± 0.4
MVO_2_ (μmol/g wet weight/min)	6.1 ± 0.3	6.7 ± 0.7	6.7 ± 0.4	6.7 ± 0.5	5.8 ± 0.1	7.6 ± 0.7

**Figure 5.**
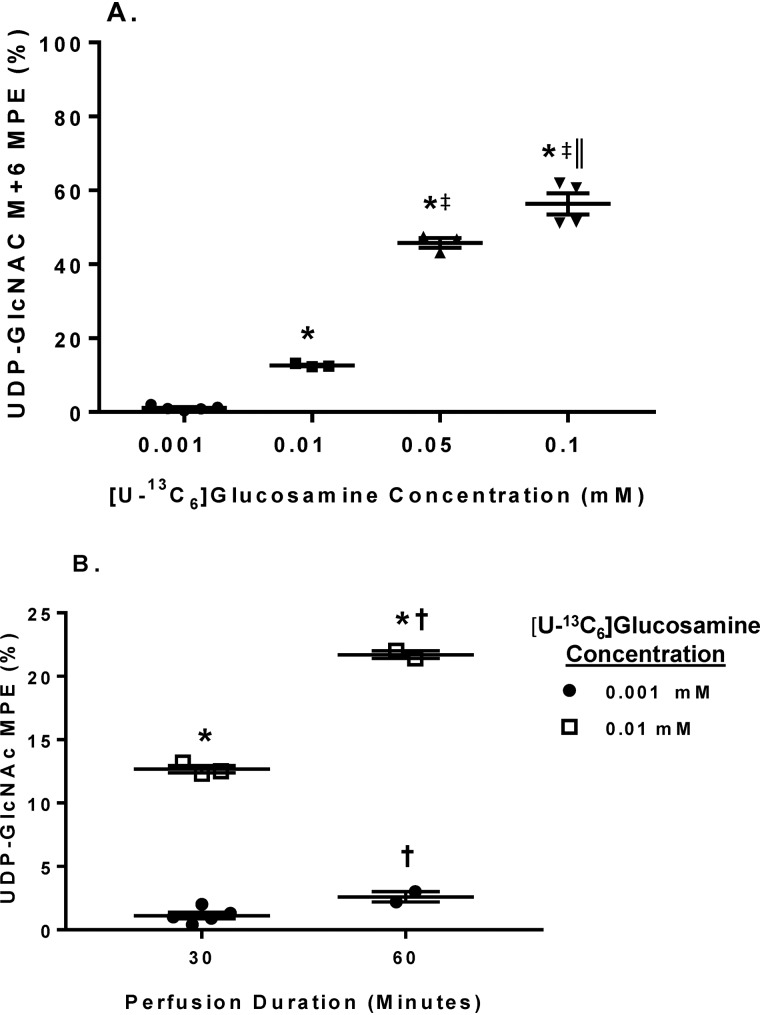
**The effect of various [U-^13^C_6_]glucosamine concentrations and perfusion durations on tissue UDP-GlcNAc M+6 MPE.** *A*, changes in UDP-GlcNAc M+6 MPE in response to increasing [U-^13^C_6_]glucosamine concentrations. *B*, effect of perfusion duration for the indicated [U-^13^C_6_]glucosamine concentrations on UDP-GlcNAc M+6 MPE. *Horizontal bars*, means ± S.E. (*error bars*). In *A*, *n* = 5 for 0.001, *n* = 3 for 0.01, *n* = 3 for 0.05, and *n* = 4 for 0.1. In *B*, *n* = 5 for 0.001 mm 30-min perfusions, *n* = 2 for 0.001 mm 60-min perfusions, *n* = 3 for 0.01 mm 30-min perfusions, and *n* = 2 for 0.01 mm 60-min perfusions. For A, *, *p* < 0.05 between 0.001 and the indicated groups; ‡, *p* < 0.05 between 0.01 and the indicated groups; ‖, *p* < 0.05 between 0.05 and 0.1. For *B*, *, *p* < 0.05 between the [U-^13^C_6_]glucosamine concentrations at the same perfusion duration; †, *p* < 0.05 for the same [U-^13^C_6_]glucosamine concentration at 60 min *versus* 30 min.

As for UDP-GlcNAc M+6 MPE, with the 30-min perfusions, UDP-GlcNAc M+6 MPE increased proportionally to [U-^13^C_6_]glucosamine concentrations at 0.001, 0.01, and 0.05 mm (Fig. 5*A*). UDP-GlcNAc M+6 MPE approximately doubled with the 60-min perfusions of 0.001 and 0.01 mm [U-^13^C_6_]glucosamine compared with their respective 30-min perfusions (Fig. 5*B*). Thus, we demonstrate the ability of our new LC-MS methods to measure UDP-GlcNAc M+6 MPE and tissue concentrations. Further, these results are consistent with glucosamine proportionally altering HBP flux and validate that changes in UDP-GlcNAc M+6 MPE and tissue concentration reflect the rate of UDP-GlcNAc synthesis.

### Impact of [U-^13^C_6_]glucose concentrations on energy metabolism and HBP flux

Our next goal was to determine whether glucose availability regulates HBP flux. To accomplish this, isolated mouse hearts were perfused in a working mode for 10, 20, 30, or 60 min with either normal or high concentrations of [U-^13^C_6_]glucose (5.5 or 25 mm, MPE = 99%) along with unlabeled physiological substrates (see “Experimental procedures” for details). Cardiac function for these perfusions is shown in Table 3. There were no significant differences in cardiac function except for modest increases in +d*P*/d*T*_max_ at 20 and 30 min in the 25 mm [U-^13^C_6_]glucose group compared with the 5.5 mm group.

**Table 3:** **Working heart function for the [U-^13^C_6_]glucose perfusions** Reported values are just prior to the end of the perfusion. Values are mean ± S.E. *, *p* < 0.05 *versus* [U-^13^C_6_]glucose 5.5 mm for the same perfusion duration.

Perfusion duration (min)	5.5 mm [U-^13^C_6_]glucose				25 mm [U-^13^C_6_]glucose			
	10 (*n* = 5)	20 (*n* = 3)	30 (*n* = 3)	60 (*n* = 2)	10 (*n* = 4)	20 (*n* = 5)	30 (*n* = 3)	60 (*n* = 2)
Heart rate (beats/min)	368 ± 27	374 ± 19	351 ± 32	409 ± 11	378 ± 23	332 ± 16	332 ± 8	344 ± 14
Developed pressure (mm Hg)	63.2 ± 2.8	60.7 ± 2.2	60.0 ± 1.5	66.5 ± 3.5	67.5 ± 5.1	66.8 ± 2.4	70.7 ± 3.7	64.0 ± 5.0
+d*P*/d*T*_max_ (mm Hg/s)	3570 ± 300	3286 ± 265	3744 ± 184	3117 ± 178	3647 ± 138	4023 ± 168*	4526 ± 361*	3720 ± 199
−d*P*/d*T*_min_ (mm Hg/s)	−3161 ± 368	−2721 ± 98	−2720 ± 589	−2631 ± 317	−2890 ± 177	−2915 ± 96	−3053 ± 109	−3160 ± 303
Aortic flow (ml/min)	8.2 ± 0.8	9.3 ± 0.2	9.1 ± 0.5	6.4 ± 2.0	10.5 ± 0.9	10.2 ± 0.4	10.1 ± 0.2	10.2 ± 0.3
Coronary flow (ml/min)	3.9 ± 1.1	2.6 ± 0.3	2.8 ± 0.5	2.2 ± 0.4	5.3 ± 1.8	2.8 ± 0.2	3.5 ± 0.4	2.7 ± 0.1
MVO_2_ (μmol/g wet weight/min)	10.8 ± 3.3	6.7 ± 0.5	7.0 ± 0.3	6.2 ± 0.8	9.1 ± 0.6	8.1 ± 0.4	8.6 ± 0.4	6.7 ± 0.9

**Figure 6.**
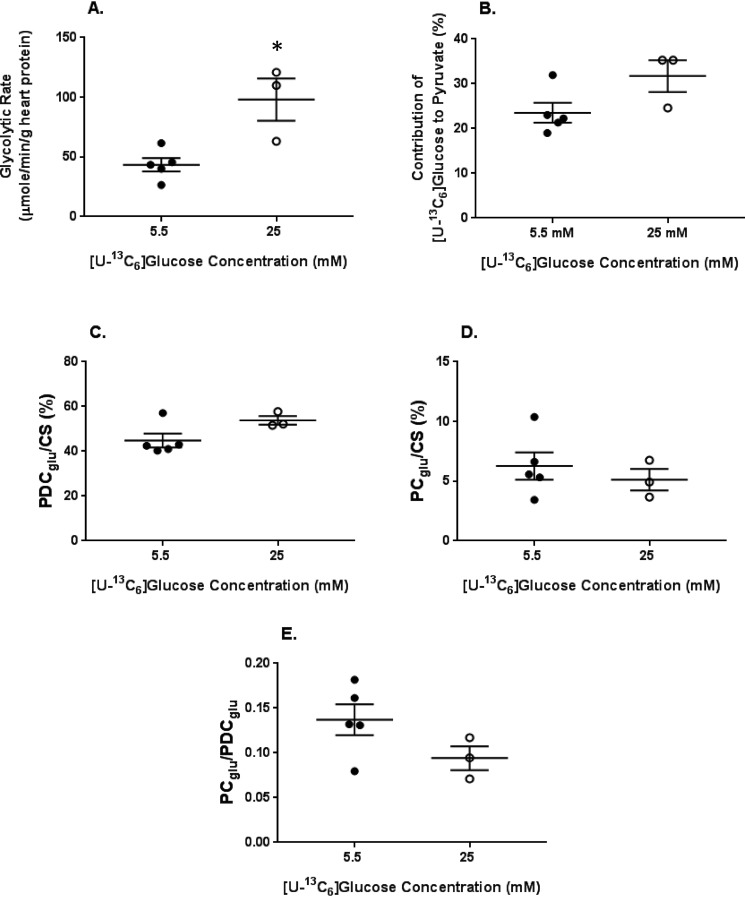
**The effect of [U-^13^C_6_]glucose concentration on metabolic fluxes relevant to energy production and the citric acid cycle.** *A*, glycolytic efflux of lactate and pyruvate formed from exogenous [U-^13^C_6_]glucose. *B*, percentage contribution of exogenous [U-^13^C_6_]glucose to the intracellular pyruvate pool. *C*, percentage contribution to acetyl-CoA formation of exogenous [U-^13^C_6_]glucose via pyruvate decarboxylation (*PDC_glu_*) relative to citrate synthesis (*CS*). *D*. Percent contribution of exogenous [U-^13^C_6_]glucose to anaplerosis via pyruvate carboxylation (*PC_glu_*) relative to CS. *E*, ratio of exogenous [U-^13^C_6_]glucose undergoing *PC_glu_* to *PDC_glu_. n* = 5 for 5.5 mm and *n* = 3 for 25 mm. *Horizontal bars*, means ± S.E. (*error bars*). *, *p* < 0.05 between the glucose concentrations. *p* = 0.08 in *B* and *p* = 0.09 in *C*.

The glycolytic rate, defined as the efflux of ^13^C-labeled lactate and pyruvate formed from exogenous [U-^13^C_6_]glucose (25), was 43.5 ± 5.6 μmol/g of heart protein/min at 5.5 mm [U-^13^C_6_]glucose and nearly doubled at 98.0 ± 17.7 μmol/g of heart protein/min with 25 mm [U-^13^C_6_]glucose (Fig. 6*A*). In the 25 mm [U-^13^C_6_]glucose perfusions, there was a small, albeit nonsignificant, increase in the relative contribution of exogenous [U-^13^C_6_]glucose to both pyruvate (*p* = 0.081; Fig. 6*B*) and acetyl-CoA formation for citrate synthesis via pyruvate decarboxylation (*PDC_glu_*/*CS*, *p* = 0.085; Fig. 6*C*) *versus* the lower glucose concentration. Pyruvate carboxylation, the anaplerotic reaction whereby pyruvate is carboxylated to form the citric acid cycle intermediate oxaloacetate, was similar between the groups (Fig. 6*D*) as was the relative ratio of the exogenous [U-^13^C_6_]glucose undergoing pyruvate carboxylation to pyruvate decarboxylation (Fig. 6*E*).

**Figure 7.**
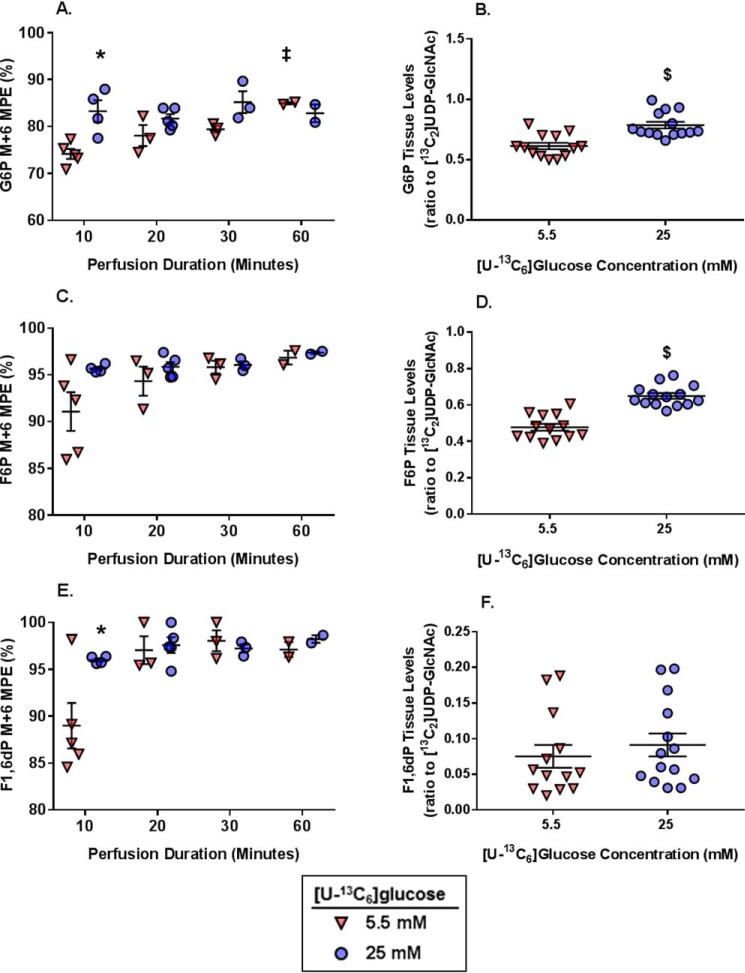
**The effect of [U-^13^C_6_]glucose concentrations and perfusion durations on ^13^C-labeling and concentration of myocardial tissue metabolites relevant to the glycolysis including precursors for the HBP.** *A*, G6P M+6 MPE. *B*, G6P tissue levels (ratio to [^13^C_2_]UDP-GlcNAc external standard). *C*, F6P M+6 MPE. *D*, F6P tissue levels (ratio to [^13^C_2_]UDP-GlcNAc external standard). *D*, F1,6dP M+6 MPE. *E*, F1,6dP tissue levels (ratio to [^13^C_2_]UDP-GlcNAc external standard). For *A*, *C*, and *E*, *n* = 5 for 5.5 mm 10-min perfusions, *n* = 4 for 25 mm 10-min perfusions, *n* = 3 for 5.5 mm 20-min perfusions, *n* = 5 for 25 mm 20-min perfusions, *n* = 3 for 5.5 mm 30-min perfusions, *n* = 3 for 25 mm 30-min perfusions, *n* = 2 for the 5.5 mm 60-min perfusions, and *n* = 2 for the 25 mm 60-min perfusions. For *B*, *D*, and *F*, the values did not change over time and are shown as combined groups with *n* = 13 for 5.5 mm and *n* = 14 for 25 mm. *Horizontal bars*, means ± S.E. (*error bars*). *, *p* < 0.05 between the groups at the same perfusion duration; ‡, *p* < 0.05 *versus* the immediately preceding perfusion duration for the same glucose concentration; $, *p* < 0.05 5.5 mm *versus* 25 mm.

We next evaluated ^13^C-labeling of G6P and F6P, which are glycolytic intermediates and precursors for the HBP. G6P M+6 MPE was greater in the 25 mm group than the 5.5 mm group at 10 min, but the MPEs were equivalent at all other perfusion durations (Fig. 7*A*). G6P M+6 MPE increased in the 5.5 mm group from 30 to 60 min. Tissue levels of G6P (relative to the external standard [^13^C_2_]UDP-GlcNAc) did not vary over time and were significantly higher in the 25 mm hearts as a group *versus* the 5.5 mm hearts (Fig. 7*B*). F6P is the next intermediate and at branch point between HBP and further glycolysis. F6P M+6 MPE was greater than 90% at the 10-min perfusion time for both glucose concentrations and stabilized at around 95% by 20 min (Fig. 7*C*). There were no significant differences in F6P M+6 MPE between glucose concentrations compared at the same perfusion duration, and F6P M+6 MPE did not increase with the longer perfusion durations. F6P tissue levels (relative to the external standard [^13^C_2_]UDP-GlcNAc) also did not vary over time and were higher in the 25 mm group compared with 5.5 mm (Fig. 7*D*). The greater tissue levels of both G6P and F6P in the 25 mm group are consistent with the higher glycolytic rate.

The first glycolytic intermediate following the HBP branch point, F1,6dP, was significantly more ^13^C-enriched at 10 min in the 25 mm *versus* 5.5 mm group (Fig. 7*E*), but these values were similar between the glucose concentrations at all other perfusion durations. There was a modest increase (*p* = 0.055) in F1,6dP M+6 MPE for the 5.5 mm group from 10 to 20 min. Tissue levels of F1,6dP (relative to the external standard [^13^C_2_]UDP-GlcNAc) were similar between the groups and did not vary over time (Fig. 7*F*); however, there was a large S.E. in both groups likely from the peak tailing of this intermediate (described above).

**Figure 8.**
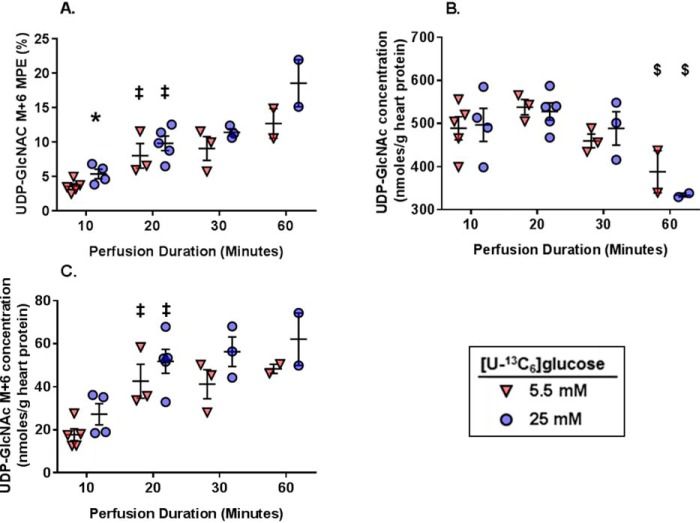
**The effect of [U-^13^C_6_]glucose concentrations and perfusion durations on ^13^C-labeling and concentration of myocardial tissue metabolites relevant to the HBP.** *A*, UDP-GlcNAc M+6 MPE. *B*, UDP-GlcNAc concentrations. *C*, UDP-GlcNAc M+6 myocardial concentrations. For all graphs, *n* = 5 for 5.5 mm 10-min perfusions, *n* = 4 for 25 mm 10-min perfusions, *n* = 3 for 5.5 mm 20-min perfusions, *n* = 5 for 25 mm 20-min perfusions, *n* = 3 for 5.5 mm 30-min perfusions, *n* = 3 for 25 mm 30-min perfusions, *n* = 2 for the 5.5 mm 60-min perfusions, *n* = 2 for the 25 mm 60-min perfusions. *, *p* < 0.05 between the groups at the same perfusion duration; ‡, *p* < 0.05 *versus* the immediately preceding perfusion duration for the same glucose concentration; $, *p* < 0.05 *versus* the 20-min perfusion duration for the same glucose concentration.

Last, we evaluated flux of [U-^13^C_6_]glucose through the HBP. We determined the tissue levels of glutamine, the amino acid required for the first step of the HBP, and found similar levels for all groups and perfusion durations (data not shown). Thus, glutamine levels should not differentially affect the ability of glucose to enter the HBP for any of the experimental groups. We subsequently focused on UDP-GlcNAc, the final product of the HBP. UDP-GlcNAc M+6 MPEs were determined over time in both the 5.5 and 25 mm [U-^13^C_6_]glucose concentrations. UDP-GlcNAc M+6 MPE significantly increased from 10 to 20 min in both glucose concentrations (Fig. 8*A*); however, there were no further increases in UDP-GlcNAc M+6 MPE with the longer perfusion durations. UDP-GlcNAc M+6 MPE was higher with 25 mm compared with 5 mm [U-^13^C_6_]glucose at 10 min; however, there were no differences at any other time points. UDP-GlcNAc tissue concentrations were similar between 5 and 25 mm groups regardless of perfusion duration; however, UDP-GlcNAc concentrations were lower at 60 min compared with the 20-min perfusions with the same glucose concentrations (Fig. 8*B*). Using the UDP-GlcNAc M+6 MPE and concentrations, we determined the absolute amount of UDP-GlcNAc generated from the exogenous [U-^13^C_6_]glucose, which will hereafter be referred to as the UDP-GlcNAC M+6 concentration. The UDP-GlcNAc M+6 concentration rose significantly from 10 to 20 min with both glucose concentrations and then plateaued with the longer perfusion times (Fig. 8*C*). To calculate glucose flux through the HBP, we used the 10- and 20-min perfusion times because total UDP-GlcNAc concentration values were stable between these times. The amount of UDP-GlcNAC M+6 produced between 10 to 20 min was calculated to be 24.9 nmol/g of heart protein with 5.5 mm [U-^13^C_6_]glucose and 24.6 nmol/g of heart protein with 25 mm [U-^13^C_6_]glucose. Thus, the rate of glucose flux through the HBP to UDP-GlcNAc between these time points was ∼2.5 nmol/g of heart protein/min with 5.5 mm glucose and 2.5 nmol/g of protein/min with 25 mm glucose. As a percentage of glycolysis, HBP flux was ∼0.006% at 5.5 mm [U-^13^C_6_]glucose and 0.003% at 25 mm [U-^13^C_6_]glucose. Overall, these results show that absolute glucose flux through the HBP remains stable even when increasing the rate of glycolysis.

### Impact of reducing cardiac workload and energetic demands on HBP flux

Flux through the HBP as a percentage of glycolysis was substantially lower than anticipated, and the flux rate was unaffected by glucose concentrations. We wondered therefore whether HBP flux was artificially inhibited during working heart perfusions because it is a non-energy-generating pathway that consumes ATP. Therefore, we tested whether lowering myocardial energetic demands would affect HBP flux by perfusing hearts retrograde into the aorta with [U-^13^C_6_]glucose and unlabeled physiologic substrates in an unloaded manner (hereafter referred to as the Langendorff mode) while beating (Beating group) or with arrest of ventricular contraction (Nonbeating group).

**Figure 9.**
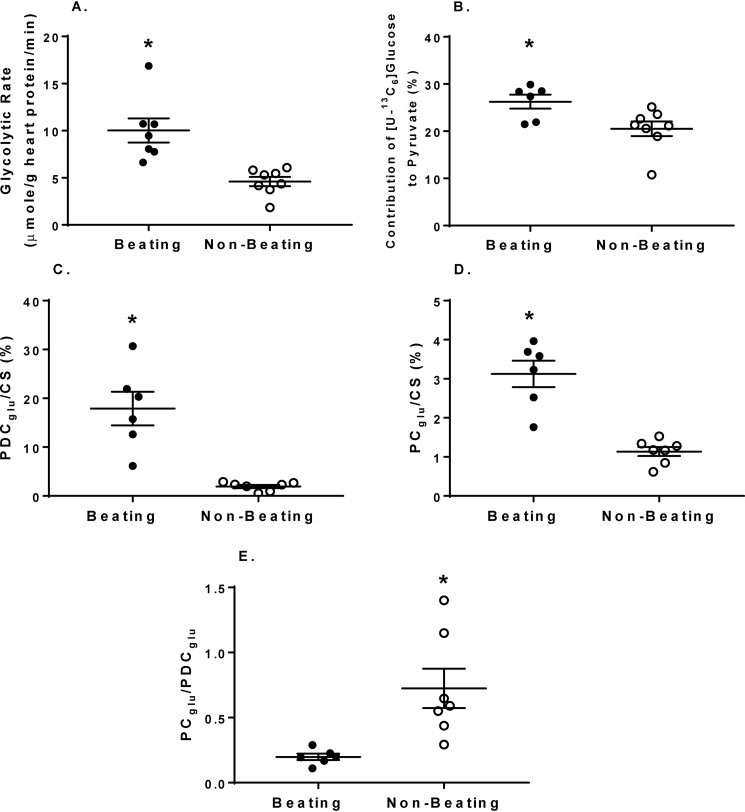
**The effect of reducing cardiac workload via Langendorff heart perfusions on metabolic fluxes relevant to energy production and the citric acid cycle.** *A*, glycolytic efflux of lactate and pyruvate formed from exogenous [U-^13^C_6_]glucose. *B*, percentage contribution of exogenous [U-^13^C_6_]glucose to the intracellular pyruvate pool. *C*, percentage contribution to acetyl-CoA formation of exogenous [U-^13^C_6_]glucose via pyruvate decarboxylation (*PDC_glu_*) relative to citrate synthesis (*CS*). *D*, percentage contribution of exogenous [U-^13^C_6_]glucose to anaplerosis via pyruvate carboxylation (*PC_glu_*) relative to CS. *E*, ratio of exogenous [U-^13^C_6_]glucose undergoing *PC_glu_* to *PDC_glu_. Horizontal bars*, means ± S.E. (*error bars*). For the Beating group, *n* = 7 in *A* and *n* = 6 for all other graphs. For the Nonbeating group, *n* = 8 for all graphs. *, *p* < 0.05 Beating *versus* Nonbeating.

Cardiac function is shown in Table 4. Oxygen consumption is a direct measure of energetic demand, and, as expected, it was markedly lower in the Beating group compared with the earlier working heart perfusions and even lower in the Nonbeating group. Consistent with this finding, the glycolysis rate was ∼50% lower in the Nonbeating *versus* Beating hearts (Fig. 9*A*), with values of 4.6 ± 0.5 μmol/g of heart protein/min (Nonbeating) and 10.0 ± 1.3 μmole/g of heart protein/min (Beating). Compared with the working heart perfusions with a similar glucose concentration (5.5 mm), the glycolytic rate was reduced by 75.6% in the Beating group and 89.4% in the Nonbeating groups. The relative contributions of [U-^13^C_6_]glucose to production of pyruvate, acetyl-CoA formation for citrate synthesis, and pyruvate carboxylation were also significantly reduced in the Nonbeating *versus* Beating hearts (Fig. 9, *B–E*).

**Table 4:** **Cardiac function for the Langendorff perfusions** Reported values are just prior to the end of the perfusion. Values are mean ± S.E. *, *p* < 0.05 *versus* Beating for the same perfusion duration.

Perfusion duration (min)	Beating			Nonbeating		
	20 (*n* = 4)	30 (*n* = 3)	40 (*n* = 4)	20 (*n* = 3)	30 (*n* = 4)	40 (*n* = 4)
Heart rate (beats/min)	361 ± 10	408 ± 40	423 ± 15	0 ± 0*	0 ± 0*	0 ± 0*
Developed pressure (mm Hg)	22 ± 4	41 ± 22	28 ± 3	0 ± 0*	0 ± 0*	0 ± 0*
+d*P*/d*T*_max_ (mm Hg/s)	1171 ± 279	1791 ± 584	1421 ± 129	0 ± 0*	0 ± 0*	0 ± 0*
−d*P*/d*T*_min_ (mm Hg/s)	−831 ± 129	−1294 ± 459	−1009 ± 167	0 ± 0*	0 ± 0*	0 ± 0*
Coronary flow (ml/min)	1.32 ± 0.08	1.76 ± 0.52	1.37 ± 0.03	1.00 ± 0.10*	0.89 ± 0.06	0.90 ± 0.06*
MVO_2_ (μmol/g wet weight/min)	3.0 ± 0.2	4.1 ± 1.2	2.7 ± 0.2	2.1 ± 0.2*	2.0 ± 0.2	2.0 ± 0.1*

**Figure 10.**
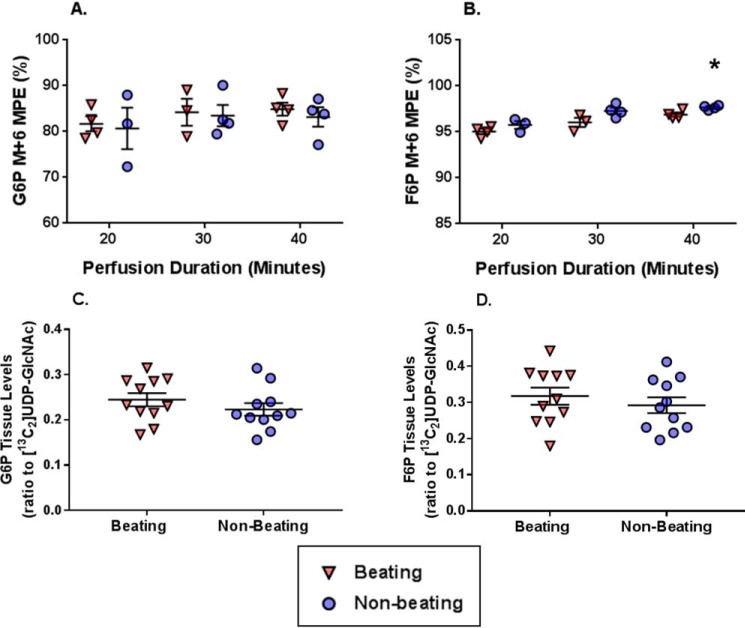
**The effect on reducing cardiac workload via Langendorff perfusions on ^13^C-labeling and concentration of metabolites relevant to glycolysis, including precursors for the HBP.** *A*, G6P M+6 molar percentage enrichment (MPE). *B*, F6P M+6 MPE. *C*, G6P tissue levels (ratio to [^13^C_2_]UDP-GlcNAc internal standard). *D*, F6P tissue levels (ratio to [^13^C_2_]UDP-GlcNAc external standard). *Horizontal bars*, means ± S.E. (*error bars*). For *A* and *B*, *n* = 4 for Beating 20 min, *n* = 3 for Nonbeating 20 min, *n* = 3 for Beating 30 min, *n* = 4 for Nonbeating 30 min, *n* = 4 for Beating 40 min, and *n* = 4 for Nonbeating 40 min. For *C* and *D*, the values did not change over time and are shown as combined groups with *n* = 11 for Beating and *n* = 11 for Nonbeating. *, *p* < 0.05 Beating *versus* Nonbeating at the same perfusion duration; ‡, *p* < 0.05 Beating *versus* Nonbeating.

G6P M+6 MPE was similar between Beating and Nonbeating groups throughout the perfusion durations (Fig. 10*A*). F6P M+6 MPE exceeded 95% for all perfusion durations (Fig. 10*B*). There was a small, but significant, increase in F6P M+6 MPE in the Nonbeating *versus* Beating groups at 40 min (97.7 ± 0.1 *versus* 96.9 ± 0.2, respectively). The tissue levels of both G6P and F6P (relative to the external standard [^13^C_2_]UDP-GlcNAc) were similar between Beating and Nonbeating groups and did not fluctuate over time (Fig. 10, *C* and *D*, respectively).

**Figure 11.**
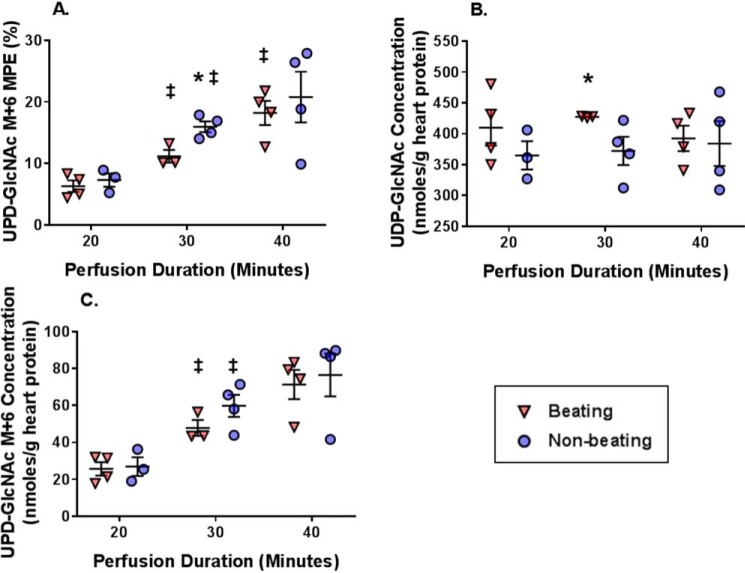
**The effect on reducing cardiac workload via Langendorff perfusions on ^13^C-labeling and concentration of metabolites relevant to the HBP.** *A*, UDP-GlcNAc M+6 MPE. *B*, UDP-GlcNAc concentrations. *C*, UDP-GlcNAc M+6 concentrations. *Horizontal bars*, means ± S.E. (*error bars*); *n* = 4 for Beating 20 min, *n* = 3 for Nonbeating 20 min, *n* = 3 for Beating 30 min, *n* = 4 for Nonbeating 30 min, *n* = 4 for Beating 40 min, *n* = 4 for Nonbeating 40 min. *, *p* < 0.05 Beating *versus* Nonbeating at the same perfusion duration; ‡, *p* < 0.05 *versus* the immediately preceding perfusion duration for the same experimental group.

UDP-GlcNAc M+6 MPE significantly increased from 20 to 30 min in both groups; however, the increase from 30 to 40 min only reached significance in the Beating group (Fig. 11*A*). Comparing the groups at the same perfusion duration, UDP-GlcNAc M+6 MPE was higher in the Nonbeating *versus* Beating group at 30 min. UDP-GlcNAc myocardial concentrations were similar between the groups for all perfusion times with the exception of 30 min. Overall, UDP-GlcNAc concentrations remained stable from 20 through 40 min (Fig. 11*B*). The calculated quantity of UDP-GlcNAC M+6 (UDP-GlcNAc M+6 concentration, Fig. 11*C*) produced between 20 and 40 min was 45.6 and 49.6 nmol/g of heart protein in the Beating and Nonbeating groups, respectively, and the corresponding estimated glucose flux through the HBP was ∼2.3 (Beating) and 2.5 (Nonbeating) nmol/g of heart protein/min. However, as the glycolytic rate differed between the groups, HBP flux as a percentage of glycolysis was ∼0.05% in the Nonbeating group and 0.023% in the Beating group. Glutamine concentrations were similar among the groups (data not shown). Overall, these results show that lowering energetic requirements and glycolytic flux did not alter the HBP flux rate.

## Discussion

Despite an increasing understanding of the diverse roles that protein *O*-GlcNAcylation plays in regulating cellular function, the inability to quantify HBP flux has greatly hindered our understanding of the factors controlling protein *O*-GlcNAc levels. Here, we have developed methods for quantifying HBP flux using ^13^C-labeled substrates and have validated these methods in the isolated perfused heart with [U-^13^C_6_]glucosamine, which bypassed the rate-limiting enzyme GFAT to directly enter the HBP. To the best of our knowledge, this is the first study to directly measure HBP flux in a metabolically active system such as the perfused heart. Whereas glucosamine perfusions demonstrated the expected increase in relative HBP flux with increasing concentrations, we found that increasing glucose concentrations, despite significantly increasing the rates of glycolysis, had no effect on the rate of UDP-GlcNAc synthesis.

We decided to use LC-MS over other methods for several reasons. First, LC-MS has increased sensitivity to detect low abundance of unlabeled and ^13^C-labeled metabolites, especially compared with ^13^C magnetic resonance spectroscopy. Second, as opposed to GC-MS, sample derivatization is not necessary with LC-MS to detect the metabolic intermediates of interest in both glycolysis and the HBP. Finally, although HPLC has been used to quantify UDP-GlcNAc (32), this technique cannot separate the various UDP-GlcNAc mass isotopomers generated from [U-^13^C_6_]glucose or other ^13^C-labeled substrates. Therefore, HPLC cannot determine the fraction of ^13^C-labeled UDP-GlcNAc generated from the ^13^C-labeled substrate *versus* other sources, such as unlabeled glucose or glycogen. The method that we have developed using LC-MS enables reproducible and accurate determination of the concentration and ^13^C-labeling of UDP-GlcNAc using LC-QToF. Our method also enables accurate measurements of the ^13^C-enrichment of the glycolytic intermediates, G6P and F6P, as well as their relative tissue concentrations expressed as a ratio to the external standard of [^13^C_2_]UDP-GlcNAc. For a precise quantitative assessment of G6P and F6P, one could use internal standards of U-^13^C_2_–labeled G6P and F6P, which can be custom-synthesized, albeit at high cost, upon special request from some companies. F1,6dP showed more peak tailing than G6P and F6P, resulting in more variability in its measurements in some experiments. Peak tailing for sugar phosphates has been previously reported by others (33, 34). We recently found that it can be circumvented by the addition of Agilent's InfinityLab Deactivator additive (Agilent Technologies (Santa Clara, CA); 1 ml/min in solvent A; data not shown). Of note, our method developed using the LC-QToF showed a similar performance using LC-triple quadrupole (data not shown), which could then be used as an alternative LC-MS instrument.

We made several noteworthy insights through the application of our new method in the isolated perfused hearts. The first was the need to use ∼99% [U-^13^C_6_]glucose MPE to reliably detect UDP-GlcNAc M+6, which is a higher [U-^13^C_6_]glucose MPE than typically required to evaluate energy metabolism (26, 35). The higher [U-^13^C_6_]glucose MPE also resulted in a F6P MPE of >90% within 10 min in the working heart perfusions, demonstrating that from this time point onward, the majority of the glucose entering the HBP pathway originates from exogenous [U-^13^C_6_]glucose. This observation provides a solid rationale for using UDP-GlcNAc M+6 measurements from perfusion durations of 10 min and longer for quantifying glucose flux through the HBP. However, it is important to note that UDP-GlcNAc concentrations decreased in the working heart at 60 min of perfusion, indicating the importance of absolute quantification for evaluating HBP flux. Additional studies are needed to understand the reason for the decrease in UDP-GlcNAc, which may yield better insights into the regulation of HBP flux. Separation of UDP-GlcNAc and UDP-GalNAc is known to be challenging, and this value is frequently reported as UDP-HexNAc (27). By fine tuning the buffer composition, we were able to separate these metabolites and found that ∼90% of the UDP-HexNAc is comprised of UDP-GlcNAc in our studies. Due to the high percentage of UDP-GlcNAc, we did not perform this additional step for most of the experiments; however, it would be important to include this step when implementing this technique in new biological systems. It is will also be important to know whether this ratio changes in response to physiological or pathological stresses.

Based on the rates of glycolysis and UDP-GlcNAc synthesis, we found that glucose metabolism via the HBP was only ∼0.006% of the glycolytic efflux, which is much lower than the frequently cited estimate of 2–3% of glucose uptake consumed by the HBP estimated from cultured adipocytes (19). It is also widely accepted that increasing glucose availability increases flux through the HBP; however, whereas increasing [U-^13^C_6_]glucose from 5.5 to 25 mm doubled the rate of glycolysis, the rates of UDP-GlcNAc synthesis were virtually identical at ∼2.5 nmol/g of heart protein/min for both groups. This also means that relative to glycolysis, the flux through the HBP decreased from 0.006 to 0.003% with the higher glucose concentration. Thus, absolute myocardial HBP flux was not affected by increased glucose availability, but the relative rate actually decreased.

We were surprised by the low rates of HBP flux and wondered whether because the HBP is a non-energy-generating pathway that consumes ATP, glucose metabolism via the HBP could be inhibited due to the high energy demands of the working heart perfusions. To address this issue, we quantified glucose metabolism via glycolysis and the HBP in the unloaded Langendorff perfused hearts, both Beating and Nonbeating. As expected, glycolytic flux and MVO_2_ were substantially lower in the Langendorff preparations compared with the working hearts and were decreased further in the Nonbeating *versus* Beating group, consistent with lower energetic demands. However, glucose flux through the HBP was similar to the working heart perfusions at ∼2.3 nmol/g of heart protein/min in the Beating group and 2.5 nmol/g of protein/min in the Nonbeating group. Thus, the high energetic demands of the working heart preparation do not appear to account for the relatively low flux of glucose through the HBP.

HBP flux was remarkably consistent across the various glycolytic rates and workloads. Thus, glucose availability does not appear to regulate flux through the HBP under these experimental conditions. We acknowledge that glucose metabolism via the HBP may be low in the perfused heart due to limitations associated with the nature of this *ex vivo* preparation, which may lack unknown factors that regulate HBP flux or artificially inhibit GFAT activity. Phosphorylation affects GFAT activity (36), and, unfortunately, technical limitations prevented us being able to determine GFAT phosphorylation in the perfused hearts. However, our results raise the possibility that the changes in glucose availability do not regulate HBP flux alone. In support of this idea, pressure overload cardiac hypertrophy augments glucose utilization and total protein *O*-GlcNAc levels; however, Nabeebaccus *et al.* (37) found that induction of GFAT1 protein by NADPH oxidase-4 was required to increase *O*-GlcNAc levels in this experimental model. HBP flux could also conceivably be a metabolic sensor for intermediates besides glucose as this pathway also requires glutamine, acetyl-CoA, and ATP. Additional experiments are clearly necessary to determine the factors regulating HBP flux in the heart and other organs using the methods described here.

Although HBP flux was similar in the Langendorff and working heart perfusions, relative glucose utilization for the HBP was higher in Langendorff perfusions at roughly 0.05% (Nonbeating) and 0.023% (Beating); however, these differences were due to the lower glycolytic rates compared with the working heart preparations. Nevertheless, it is worth noting that if the HBP flux was indeed ∼2% of the glycolytic rate in our working heart perfusions, then glucose flux via the HBP would be between 860 and 1960 nmol/g of heart protein/min for the 5 and 25 mm groups, respectively. Considering that the myocardial UDP-GlcNAc concentration averaged about 425 nmol/g of heart of protein, UDP-GlcNAc would completely turn over every 13–30 s. We believe that such a rapid turnover of UDP-GlcNAc is unlikely because it would be nearly as fast as myocardial ATP turnover. This finding highlights the limitations of expressing HBP flux relative to the glycolytic rate, particularly in biological systems with high energy demands. Consequently, given the high metabolic rate of the heart, we urge caution in projecting the relative HBP flux values reported here to other organs or cell culture models.

In conclusion, using ^13^C-labeled substrates combined with LC-MS, we have developed a method to quantify both absolute HBP flux and HBP flux relative to glycolysis in the isolated perfused mouse heart. Using [U-^13^C_6_]glucosamine, we were able to demonstrate concentration-dependent changes in UDP-GlcNAc M+6 MPE, consistent with increased HBP flux. Surprisingly, we found that changing glucose concentration from 5.5 to 25 mm had no effect on the rate of UDP-GlcNAc synthesis from exogenous glucose; moreover, this rate did not change over a wide range of workloads. The absolute HBP flux remained at ∼2.3–2.5 nmol/g of heart protein/min regardless of perfusion conditions; however, as a fraction of glycolysis, the rate varied from 0.006 to 0.023%, due to changes in the rate of glycolysis. These results indicate that, in the isolated perfused heart, glucose utilization via the HBP relative to glycolysis is much lower than that estimated in cultured adipocytes, and we propose that this is in part due to the high rates of glycolysis and energetic demands of the heart.

## Experimental procedures

### Chemicals

LC-MS grade acetonitrile was purchased from Fisher. Ammonium acetate, ammonium formate, UDP-GlcNAc, UDP-GalNAc, ammonium hydroxide (NH_4_OH; 5 m), G6P, F6P, octanoic acid, docosapentaenoic acid, glucose, glucosamine hydrochloride, oleic acid, sodium l-lactate, sodium pyruvate, l-carnitine, l-glutamine, insulin from bovine pancreas, sodium chloride, sodium bicarbonate, potassium chloride, calcium chloride, magnesium chloride, and potassium phosphate monobasic were obtained from Sigma-Aldrich. [1,2-^13^C_2_]UDP-GlcNAc was from Omicron Biochemicals (South Bend, IN), and l-[U-^13^C_5_,^15^N_2_]glutamine was from CIL (Cambridge Isotope Laboratories, Andover, MA). Water for the LC-MS and GC-MS was purified by a Milli-Q system (Millipore, Montreal). BSA fraction V-fatty acid-free grade was from Gemini Bio-Products (West Sacramento, CA). [U-^13^C_6_]glucose (MPE = 99%) was from Cambridge Isotope Laboratories, Inc. (Tewksbury, MA). d-[U-^13^C_6_]Glucosamine hydrochloride (MPE = 99%) was from Omicron Biochemicals. Blebbistatin was from Selleck Chemicals (Houston, TX).

### Animals

Male C57BL/6J mice from the Jackson Laboratory (Bar Harbor, ME) between the ages of 3 and 5 months were used for all experiments. This investigation conforms to the Guide for the Care and Use of Laboratory Animals published by the National Institutes of Health (NIH Publication 85-23, revised 1996) and was reviewed and approved by the Office of Animal Care at Seattle Children's Research Institute.

### Ex vivo heart perfusions in the working and Landendorff mode

Working heart experiments were performed as described previously (28–31). For these experiments, time 0 began following a 5-min stabilization period after starting the working mode perfusion (antegrade left atrial perfusion). For the Langendorff perfusion, heart isolation was similar to the working heart perfusions. These hearts were initially stabilized with ∼5 min of retrograde aortic perfusions with unlabeled metabolic substrates. Subsequently, the perfusion solution was switched to the ^13^C-labeled and unlabeled substrates described below with a perfusion pressure of 80 mm Hg via a gravity-fed apparatus. An SPR-PV catheter (SPR-869 or -839 Millar Pressure-Volume Systems, Millar Instruments, Inc., Houston, TX) was inserted into the left ventricle to measure cardiac function.

### Perfusion solutions

The following perfusion solution was used for the glucosamine perfusions: [U-^13^C_6_]glucosamine at the concentrations noted under “Results” (MPE = 99%) or unlabeled glucosamine, 5.5 mm glucose, 0.4 mm oleate bound to 0.75% (w/v) delipidated BSA, 0.2 mm pyruvate, 1.5 mm lactate, 50 μm carnitine, 0.5 mm glutamine, 50 microunits/liter insulin mixed in physiologic salt solution (PSS) (31).

Pilot studies were done to assess [U-^13^C_6_]glucose labeling of the HBP and glycolytic intermediates of interest with hearts perfused in a working mode with the following: 10 mm [U-^13^C_6_]glucose along with PSS, 0.7 mm oleate bound to 3% (w/v) delipidated BSA, 0.8 nm insulin, 0.2 mm pyruvate, 1.5 mm lactate, 50 μm carnitine, 0.5 mm glutamine, and 1 μm glucosamine. Subsequently, to assess the impact of glucose availability on glucose flux through the HBP, hearts were perfused with [U-^13^C_6_]glucose (5.5 mm or 25 mm, MPE = 99%), along with PSS, 0.7 mm oleate bound to 3% (w/v) delipidated BSA, 0.8 nm insulin, 0.2 mm pyruvate, 1.5 mm lactate, 50 μm carnitine, 0.5 mm glutamine, and 1 μm glucosamine. To arrest ventricular contraction in the Nonbeating group, the perfusion solution also included blebbistatin (10 μm; Selleck Chemicals, Houston, TX).

### Western blotting

Western blotting was performed as described previously on freshly isolated protein from perfused hearts or *in vivo* isolated controls with the RL-2 antibody to determine total protein *O*-GlcNAc levels (4, 29–31). We attempted Western blot analysis for GFAT1/2 and phosphorylated GFAT1; however, the albumen from the heart perfusions interfered with the signal intensity of these proteins.

### MS-based analysis of metabolite concentration and ^13^C-enrichment for metabolic flux analysis

#### Sample collection

Procedures for sample collection and processing for metabolite analysis in perfusates and heart tissues were performed as described previously (25).

#### Measurements of substrate fluxes relevant for energy production

Previously published studies (25, 38, 39) provide definitions of the ^13^C terminology and descriptions for measurements by GC-MS (Agilent 6890N GC coupled to a 5973N MS) and equations for the calculations of flux ratios relevant to substrate selection for energy production through mitochondrial citrate synthesis from the ^13^C-enrichment of the acetyl (carbons 4 and 5) and oxaloacetate (carbons 1, 2, 3, and 6) moiety of citrate. Previous studies have shown that it takes approximately a 30-min perfusion time to reach steady state for flux measurements of the citric acid cycle; therefore, these measurements were only made for hearts perfused for 30 min or longer. Influent and effluent perfusates were used to assess ^13^C-enrichment in lactate and pyruvate arising from cytosolic glycolysis of exogenous [U-^13^C_6_]glucose using GC-MS by a previously published method (25, 38, 39), and this measurement is referred to as the glycolytic rate throughout the paper.

#### HBP flux

These techniques will be described in detail as they are new. Freeze-clamped heart tissues were used to assess the concentration and ^13^C-labeling of metabolites relevant to the assessment of HBP flux, namely G6P, F6P, F1,6dP, and UDP-GlcNAc as well as in some perfusions of UDP-GalNAc, using the newly developed and validated LC-MS methods described below.

##### Extraction

Samples of 50 mg of freeze-clamped heart tissue powder, which had been pulverized in liquid nitrogen, were deproteinized with a mix of 1 ml of 70% methanol, added to 20 μl of the internal standard [^13^C_2_]UDP-GlcNAc 0.2 mm (4 nmol) and [U-^13^C_5_,^15^N_2_]glutamine (150 nmol) for 10 min on ice and homogenized for 45 s using a Bead Ruptor 12 homogenizer (Omni International, Kennesaw, GA) with six beads (2.8-mm ceramic bead medium, Omni International) at high intensity. Following centrifugation at 12,000 × *g* for 10 min at 4 °C, the liquid phase was collected, and the pellet was extracted a second time in 450 μl of 70% methanol. After vortex and centrifugation, the two liquid phases were combined and filtered through a 13-mm GD/X 0.45-μm syringe filter (Whatman^TM^, GE Healthcare), and the filters were washed with 1 ml of 70% methanol. The combined methanol phases were evaporated under nitrogen until 200 μl and kept at 4 °C overnight. The next morning, samples were centrifuged for 5 min at 3000 × *g*, and a volume of 2 μl of the upper liquid phase was injected into the LC-MS.

##### LC-MS analysis of UDP-GlcNAc, glutamine, G6P, F6P, and F1,6dP

This analysis was performed on a 1290 Infinity HPLC equipped with a SeQuant ZIC-pHILIC PEEK-coated column (2.1 × 150 mm, 5 μm; EMD Millipore, Billerica, MA) and coupled with a 6530 accurate mass quadrupole-TOF (LC-QToF, Agilent Technologies) with a Dual Agilent Jet Stream ESI source. Other than an inline filter of 0.3 μm, all switching valves and stainless steel connectors from the LC-MS were bypassed. Chromatographic conditions were as follows: temperature was set at 15 °C, flow rate started at 0.15 ml/min and ended at 0.125 ml/min, the mobile phase A consisted of 10 mm ammonium acetate, pH 8.75 (5 m NH_4_OH) in water, and B consisted of acetonitrile. The solvent program was 0–2 min, 80% B; 2–13 min, 80–40% B; 13–14 min, 40–30% B; 14–20 min, 30% B with flow rate 0.15 to 0.125 ml/min; 20–20.5 min, 30–0% B; 27–28 min, 0–80% B and 0.125 to 0.15 ml/min, and then an equilibration time of 9 min. LC-MS operating conditions were as follows: gas source temperature, 325 °C; drying gas rate, 13 liters/min; sheath gas temperature, 350 °C; sheath gas flow, 11 liters/min; nebulizer, 60 p.s.i.; capillary voltage, 3500 V; and nozzle voltage, 1500 V. Mass correction was applied during LC-MS analysis using on-line infusion of octanoic and docosapentaenoic acid as reference compounds. Mass spectra were acquired from *m*/*z* 130 to 625 in MS scan negative mode. MS scans were collected during 1 s. Mass spectra were acquired from *m*/*z* 130 to 625 in MS scan negative mode. MS scans were collected during 1 s. The *m*/*z* for the various metabolites are as follows: UDP-GlcNAc, 606.0743 (unlabeled), 608.0815 (M+2), and 612.0944 (M+6); Gln, 145.06119 (unlabeled) and 152.0727 (M+7); G6P, 259.0224 (unlabeled) and 259.0224 (M+6); F6P, 259.0224 (unlabeled) and 259.0224 (M+6); and F1,6dP, 338.9888 (unlabeled) and 345.0089 (M+6).

This method has been validated for linearity, limit of detection, and intra- and interday reproducibility. Linearity was tested using standard solutions of UDP-GlcNAc (0–250 μm or 0–50 nmol in 200 μl; 12 different concentrations) in nonextracted vials and heart tissue samples (50 mg; added to increasing concentrations of [^13^C_2_]UDP-GlcNAc; range 0–50 nmol or 0–250 μm). The linearities for quantification of UDP-GlcNAc, glutamine, G6P, F6P, and F1,6dP in heart tissue samples were assessed by extracting five different heart tissue samples (ranging from 20 to 70 mg of pulverized powder; *n* = 4/condition), which had been added to a constant concentration of [^13^C_2_]UDP-GlcNAc (4 nmol) and [^13^C_5_,^15^N_2_]glutamine (150 nmol). Limit of detection was tested by spiking heart tissue (50 mg) with 0.01–0.1 nmol of [^13^C_2_]UDP-GlcNAc (*n* = 3). Intra- and interday reproducibility was assessed for quantification of UDP-GlcNAc and glutamine using their respective internal standards [^13^C_2_]UDP-GlcNAc and [^13^C_5_,^15^N_2_]glutamine, respectively, and for the semiquantification of F6P, G6P, and F1,6dP using the external [^13^C_2_]UDP-GlcNAc by extracting 50 mg of pulverized heart samples (*n* = 8) over 3 different days.

##### LC-MS separation of UDP-GlcNAc and UDP-GalNAc

UDP-GlcNAc and UDP-GalNAc have similar chemical properties and were not separated using chromatographic conditions described above. To assess the amount of UDP-GalNAc contributing to the UDP-GlcNAc peak, we used the following method. Samples (2 μl) were injected on a 1290 Infinity HPLC equipped with an Agilent Zorbax RX-Sil (4.6 × 150 mm, 5 μm) and coupled with a 6530 accurate LC-QToF (Agilent Technologies) with a Dual Agilent Jet Stream ESI source. Chromatographic conditions were as follows. Temperature was set at 30 °C, and the flow rate started at 0.3 ml/min. Mobile phase A consisted of 5 mm ammonium acetate and 5 mm ammonium formate in water, and B was acetonitrile. The solvent program was as follows: 0–2 min, 95% B; 2–11 min, 95–50% B; 11–17 min, 50% B; 17–18 min, 80–95% B, and then an equilibration time of 7 min. LC-MS operating conditions were as follows: gas source temperature, 290 °C; drying gas rate, 11 liters/min; sheath gas temperature, 375 °C; sheath gas flow, 12 liters/min; nebulizer, 35 p.s.i.; capillary voltage, 3500 V; nozzle voltage, 500 V. Mass spectra were acquired from *m*/*z* 130 to 650 in MS scan negative mode. MS scans were collected during 1 s. Retention times were as follows: UDP-GlcNAc, 14.43 min; UDP-GalNAc, 14.58 min. Using this method, the percentage contribution of UDP-GalNAc to UDP-GlcNAc peak was evaluated in [U-^13^C_6_]glucose-perfused hearts (*n* = 4) and in control (nonperfused) hearts (*n* = 4).

##### LC-MS data processing

MS signals were extracted using Mass Hunter Quantitative Analysis version B.07 from Agilent. ^13^C-Labeling of UDP-GlcNAc, F6P, and G6P were quantified by extracting the MS signals corresponding to the M and M+6 ions and expressed as MPE. Tissue concentrations of (i) UDP-GlcNAc and UDP-GalNAc were quantitated using the internal standard [^13^C_2_]UDP-GlcNAc and (ii) glutamine using the internal standard of [^13^C_5_,^15^N_2_]glutamine. Tissue levels of G6P, F6P, and F1,6dP are expressed as ratios relative to the external standard of [^13^C_2_]UDP-GlcNAc.

### Protein quantification

Hearts develop edema during perfusion. Further, excess perfusion solution is often frozen with the heart during rapid freeze clamping. Thus, the measured weights of freeze-clamped hearts after perfusion are unreliable. To control for this issue, all hearts had their protein quantified by the bicinchoninic acid protein assay method. The protein concentration was used to normalize values of UDP-GlcNAc concentration and glycolytic efflux.

### Statistical analysis

All reported values are mean ± S.E. A two-way, unpaired *t* test was used for comparisons of two groups that did not involve multiple time points. For comparisons of multiple groups or two groups with multiple perfusion durations, we utilized either a one- or two-factor analysis of variance as appropriate. If the global test for the analysis of variance was statistically significant, then we performed follow-up pairwise comparisons between the groups of interest identified *a priori* by pairwise *t* tests. Because of the low number of replicates per group in some groups, we did not correct for multiple comparisons because a correction procedure would increase the likelihood of committing a type II error. This approach is consistent with the literature for exploratory studies such as ours (40–42). The criterion for significance was *p* < 0.05. We performed the statistical analysis using GraphPad Prism version 7.03 (GraphPad Software, San Diego, CA).

## Author contributions

A. K. O., B. B., J. C. C., and C. D. R. conceptualization; A. K. O. and C. D. R. resources; A. K. O., B. B., W. Z. Z., J. C. C., and C. D. R. data curation; A. K. O., B. B., W. Z. Z., J. C. C., and C. D. R. formal analysis; A. K. O. supervision; A. K. O. funding acquisition; A. K. O. validation; A. K. O., B. B., W. Z. Z., and C. D. R. investigation; A. K. O., B. B., W. Z. Z., J. C. C., and C. D. R. methodology; A. K. O., B. B., J. C. C., and C. D. R. writing-original draft; A. K. O. and C. D. R. project administration; B. B., J. C. C., and C. D. R. writing-review and editing.

## Footnotes

2 The abbreviations used are:

HBP: hexosamine biosynthesis pathway
MPE: molar percentage enrichment
OGT: *O*-GlcNAc transferase
F6P: fructose 6-phosphate
GFAT: l-glutamine:fructose-6-phosphate amidotransferase
G6P: glucose 6-phosphate
F1,6dP: fructose 1,6-diphosphate
LC-QToF: liquid chromatography-quadrupole time-of-flight
CV: coefficient of variation
UDP-HexNAc: UDP-*N*-acetylhexosamines
+d*P*/d*T*_max_: maximum derivative of pressure over time
−d*P*/d*T*_min_: minimum derivative of pressure over time
MVO_2_: myocardial oxygen consumption
PSS: physiologic salt solution.
